# Toward Probabilistic Diagnosis and Understanding of Depression Based on Functional MRI Data Analysis with Logistic Group LASSO

**DOI:** 10.1371/journal.pone.0123524

**Published:** 2015-05-01

**Authors:** Yu Shimizu, Junichiro Yoshimoto, Shigeru Toki, Masahiro Takamura, Shinpei Yoshimura, Yasumasa Okamoto, Shigeto Yamawaki, Kenji Doya

**Affiliations:** 1 Neural Computation Unit, Okinawa Institute of Science and Technology Graduate University, Onna-son, Okinawa, Japan; 2 Graduate School of Information Science, Nara Institute of Science and Technology, Ikoma, Nara, Japan; 3 Department of Psychiatry and Neurosciences, Hiroshima University, Hiroshima, Japan; 4 Faculty of Psychology, Otemon Gakuin University, Ibaraki, Osaka, Japan; Queensland Institute of Medical Research, AUSTRALIA

## Abstract

Diagnosis of psychiatric disorders based on brain imaging data is highly desirable in clinical applications. However, a common problem in applying machine learning algorithms is that the number of imaging data dimensions often greatly exceeds the number of available training samples. Furthermore, interpretability of the learned classifier with respect to brain function and anatomy is an important, but non-trivial issue. We propose the use of logistic regression with a least absolute shrinkage and selection operator (LASSO) to capture the most critical input features. In particular, we consider application of group LASSO to select brain areas relevant to diagnosis. An additional advantage of LASSO is its probabilistic output, which allows evaluation of diagnosis certainty. To verify our approach, we obtained semantic and phonological verbal fluency fMRI data from 31 depression patients and 31 control subjects, and compared the performances of group LASSO (gLASSO), and sparse group LASSO (sgLASSO) to those of standard LASSO (sLASSO), Support Vector Machine (SVM), and Random Forest. Over 90% classification accuracy was achieved with gLASSO, sgLASSO, as well as SVM; however, in contrast to SVM, LASSO approaches allow for identification of the most discriminative weights and estimation of prediction reliability. Semantic task data revealed contributions to the classification from *left precuneus, left precentral gyrus, left inferior frontal cortex (pars triangularis),* and *left cerebellum (c* *rus1)*. Weights for the phonological task indicated contributions from *left inferior frontal operculum, left post central gyrus, left insula, left middle frontal cortex, bilateral middle temporal cortices, bilateral precuneus, left inferior frontal cortex (pars triangularis),* and *left precentral gyrus*. The distribution of normalized odds ratios further showed, that predictions with absolute odds ratios higher than 0.2 could be regarded as certain.

## Introduction

Major depressive disorder (MDD) belongs to the mental, neurological, and substance-abuse diseases (MNS) currently regarded as significant challenges in global mental health [1]. Due to the complexity and variety of symptoms, MDD diagnosis requires time-consuming interviews, which rely heavily on the clinical experience of the doctor, as well as on patient cooperation. Objective diagnosis methods based on biological markers have yet to be found. The aim of this study is to corroborate development of a diagnostic method for MDD and other mental disorders by applying machine learning algorithms to functional brain imaging (fMRI) data.

Recent imaging studies show that task-related brain activation of MDD patients, as well as brain activation during rest, differs significantly from that of healthy controls [2–6], thus encouraging the diagnosis of MDD from brain imaging data using statistical machine learning algorithms (see e.g. [5, 7]). This idea is supported by the emerging field of computational psychiatry, which emphasizes the integrative, explanatory role of computational ideas in neuroscience and the impact it could have on assessing mental illnesses [8].

However, a major obstacle in applying statistical machine learning methods to brain imaging data is that the dimension of input variables (voxels) considerably exceeds the number of training samples (subjects), resulting in over-fitting. Often Support Vector Machine (SVM) [7, 9–11] is used to overcome this problem. It can largely prevent over-fitting using the principle of large margin separation [12, 13]. However, a shortcoming of SVM lies in the assignment of weights to all input features. The discriminative relevance of individual features (here, neural activation in each voxel of the imaging data) is difficult to interpret. A further limitation of deterministic classification algorithms such as SVM, is that they do not provide a measure of classification reliability. This has also been pointed out in Nouretdinov et al. [14].

In consideration of the above, we propose the application of sparse logistic regression with a least absolute shrinkage and selection operator (LASSO [15, 16]). Standard LASSO limits the number of effective variables through regularization of the L1 norm of the attributed weights. The regularization penalty is controlled through a regularization parameter (λ_*S*_).

Standard LASSO finds solutions based on a minimum number of features, but allows for selection of isolated voxels. This compromises robustness with respect to local variation in inter-subject brain function. As previous functional brain imaging studies have shown, brain activity in certain brain areas such as thalamus and frontal lobe is altered in depression patients [6, 17]. We aim to extract brain regions, rather than individual voxels. For this purpose, we propose the use of group LASSO [18], which constrains groups of features. Regularization of the number of groups is thereby subject to the Euclidian norm of weights in each group. As with standard LASSO, regularization strength can be controlled by a parameter (λ_*G*_). This approach facilitates interpretation of the learned classifier, since the remaining features inherently comprise groups. Defining voxel groups according to known functional and anatomical brain areas, we expect group LASSO to reveal brain areas critical for depression diagnosis.

Feature-wise regularization and group-wise regularization can be employed at the same time, so that the number of voxel groups (brain areas), as well as the number of individual voxels, is sparsified. The algorithm hence depends on a pair of regularization parameters (λ = (λ_*S*_, λ_*G*_)) and is then referred to as sparse group LASSO.

We verified the performance of group LASSO (gLASSO), and sparse group LASSO (sgLASSO) in comparison to that of standard LASSO (sLASSO) in the analysis of fMRI data obtained from depression patients and age-matched healthy control subjects. Since executive dysfunction is a neuropsychological constituent of depression [19], fMRI experiments were based on semantic and phonological verbal fluency tasks, in which depression patients are known to perform poorly [10, 20–23]. Moreover, Bom de Araujo et al. [24] have demonstrated that disease severity has a direct impact on verbal fluency, regardless of age, educational level, or gender. This fact is beneficial for unbiased population results. We also compared LASSO results to those of SVM [12, 13] and the Random Forest algorithm [25].

## Materials and Methods

This study was approved by the Research Ethics Committee at the Okinawa Institute of Science of Technology as well as the Research Ethics Committee of Hiroshima University (permission nr. 172). Written consent was obtained from all subjects participating in the study (approved by the Research Ethics Committee of the Okinawa Institute of Science and Technology and the Research Ethics Committee of Hiroshima University).

### Subjects

Thirty-one drug naive, i.e. first time diagnosed, patients (age 26–63, average 38:81 ± 9:76, 16 male) with major depression disorders were recruited by the Psychiatry Department of Hiroshima University and collaborating medical institutions, based on the Mini-international neuropsychiatric interview (M.I.N.I [21]), which enables doctors to identify psychiatric disorders according to the Diagnostic and Statistical Manual of Mental Disorders, Fourth Edition (DSM-IV [26]). As a control group, 31 persons (ages 23–63, average 33.45 ± 12, 15 male) with no history of mental or neurological disease, were recruited by advertisement in local newspapers. All controls underwent the same self-assessment and examinations administered to the test group. Subjects of both groups completed the Japanese version of the National Adult Reading Test [27].

### Data Acquisition & Task

fMRI measurements were performed at Hiroshima University on a 3T GE Signa HDx scanner with a 2D EP/GR (TR = 3s, TE = 27ms, FA = 90deg, matrix size 64 × 64 × 32, voxel size 4 × 4 × 4mm, no gap, interleaved). As mentioned above, subjects underwent two-block designed verbal fluency tasks known to pose difficulties for depressive patients. Structural T1 images were acquired after the fMRI experiments for correction of head position changes in the subsequent analysis (IRP FSPGR, TR = 6.824ms, TE = 1.9ms, FA = 20deg, FOV 256mm, matrix size 256 × 256 × 180, voxel size 1 × 1 × 1mm).

#### Semantic Verbal Fluency

**Fig 1:**
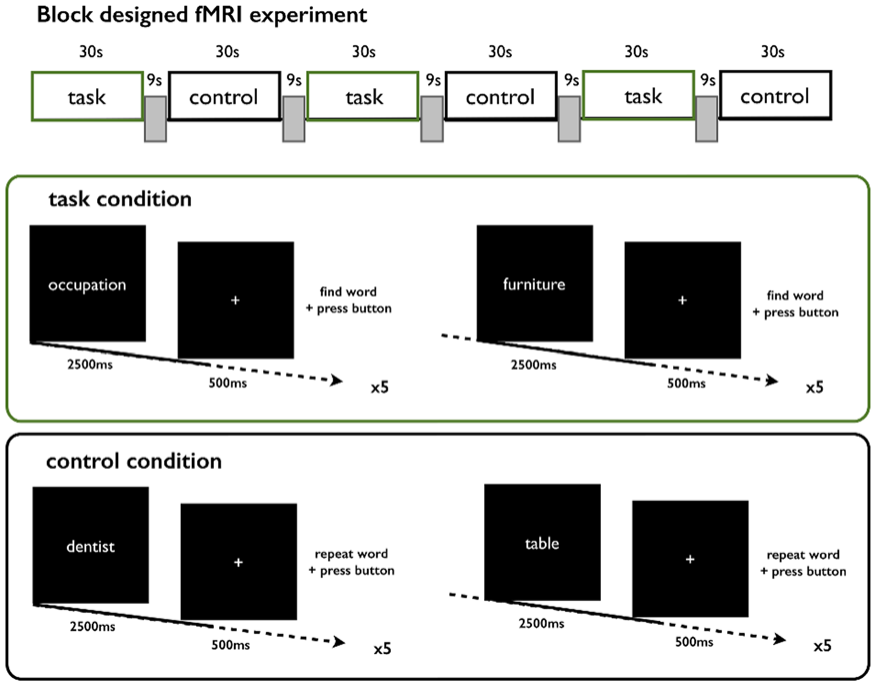
fMRI Semantic Verbal Fluency Task. The experiment consisted of three task and three control blocks. In the task condition, subjects were presented with a categorical word (semantic task) or syllable of the Japanese alphabet (phonological task) for 2500ms (e.g. occupation) for which they had to find a matching word, utter it in their minds and press a button. A white cross displayed for 500ms indicated the end of the trial. This was repeated five times each for two words referring to two different categories in the semantic task and two different syllables in the phonological task. In the control condition, the button press occurred after repeating the displayed word in the mind. Two different words were displayed five times each. The end of each block was indicated by 9 seconds of blank screen.

After an initial rest period of 30 seconds, a categorical word (e.g., furniture) was presented to each participant for 2500ms (Fig 1). A fixation cross was presented for the next 500ms. Subjects were asked to find a word matching the given category (e.g., table) and press a button once they had uttered the chosen word in their minds. After five consecutive trials repeating the same categorical word, five trials employing a different categorical word followed. Under control conditions, subjects were presented two words selected from a certain category (e.g., table), five times each. Subjects had to repeat each word in their minds and were again asked to press a button once they had done so. Nine seconds of blank screen indicated the end of the task and control blocks. This whole sequence was repeated three times and required approximately four minutes, during which 94 volumes of the whole brain were acquired.

#### Phonological Verbal Fluency

The setting of the phonological verbal fluency task was identical to that of the semantic task. Instead of categorical groups, a syllable of the Japanese alphabet was presented. Subjects then had to think of a word beginning with that syllable and to repeat it in their minds. Subjects were asked to press a button immediately thereafter.

### Data Preprocessing

For each subject, the first five volumes of each time series were discarded so as to allow for magnetic field equilibrium. The remaining volumes were processed with SPM8 (Wellcome Trust Centre for Neuroimaging, UCL, London), using following standard procedure: After slice timing correction, motion correction, co-registration to anatomical MRI, normalization with standard brain template, and smoothing (kernel width 8 × 8 × 8mm), a model conforming to the task was specified and the contrast between task and control conditions was evaluated. Z-scores that exceeded the absolute value of 5 were considered outliers and corresponding voxels were discarded from the dataset. Z-scores of voxels which could not be labeled by Automated Anatomical Labeling software (AAL [28]), provided by SPM8, were also excluded from the analysis. Vectorized subject volumes were assembled to a matrix of size 62 × 65280, in which a row represented brain activity Z-scores in the voxels of a given subject. Columns (voxels) that were zero for all subjects were discarded, leaving a data matrix of size 62 × 14055 (= number of subjects × number of remaining voxels). In the following, we refer to the remaining voxels as features and the resulting matrix as the feature matrix. The latter was normalized voxel-wise and served as input for all classification algorithms applied in this study, LASSO regressions as well as SVM and Random Forest algorithms. For combined semantic and phonological data, we simply concatenated both feature matrices.

For group LASSO, we defined voxels belonging to the same brain area as a group. Brain areas themselves were determined and labeled using the MNI standard brain template provided by the AAL toolbox. Out of 116 brain areas defined in this atlas, fMRI data covered 105 areas. Areas not covered by data were as follows: *bilateral superior frontal orbital gyri*, *bilateral middle frontal orbital gyri*, *left olfactory gyrus*, *left medial frontal orbital gyrus*, *bilateral rectus, left middle temporal pole* and *bilateral cerebellum (pars10)*. For the combined verbal fluency data we therefore arrived at 210 groups (105 brain areas for each dataset).

### Classification Algorithms

For classification, we considered three variants of logistic regression using LASSO [15, 16], SVM [12], and Random Forest [25].

#### LASSO logistic regression

Let **x**_*i*_ = (*x*_*i*1_, ⋯, *x*_*id*_) ∈ ℝ^*d*^ be the vector of values representing brain activation in each voxel of the *i*-th subject (hereafter referred to as feature vector, *i* = 1 ⋯ *n*, *n* the number of subjects). The binary label *y*_*i*_ ∈ {±1} indicates whether the subject belongs to the control (*y*_*i*_ = −1) or the patient group (*y*_*i*_ = +1). Logistic regression predicts the probability of the label *y* from the corresponding feature vector **x**, and is defined as

$$\begin{array}{c} P\left(y|x;w\right)=\frac{1}{1+\exp[-yx^{T}w]}, \end{array}$$ (1)

where **x** ^T^ is the transpose of the vector **x**. **w** = (*w*_1_, ⋯, *w*_*d*_) ∈ ℝ^*d*^ is a vector representing the contribution weights of each element in the vector **x**, and determined such that it fits the given dataset *D* = {(**x**_*i*_, *y*_*i*_)|*i* = 1, ⋯, *n*}. This can be achieved by minimizing the negative mean log-likelihood:

$$\begin{array}{c} f\left(w\right)=-\frac{1}{n}\sum_{i=1}^{n}\log P\left(y_{i}|x_{i};w\right). \end{array}$$ (2)

However, the minimizer is not well defined for *d* ≫ *n*, as is the case in our study.

The Least Absolute Shrinkage and Selection Operator (LASSO) [15, 16] is a regularization technique to restrict the number of non-zero elements in the minimiser and to make the solution unique. Consider *G* partitions on **x**, then the weight vector **w** is determined so as to minimize

$$\begin{array}{c} J\left(w\right)=f\left(w\right)+\lambda_{S}{\left\|w\right\|}_{1}+\lambda_{G}\sum_{g=1}^{G}\sqrt{\sum_{j=1}^{d}{ℐ}_{g,j}w_{j}^{2}}, \end{array}$$ (3)

where $\left\|\text{w}{\|}_{1}=\sum_{j=1}^{d}|w_{j}\right|$ and ℐ_*g*,*j*_ is the indicator function such that ℐ_*g*,*j*_ = 1 if the *j* − *th* voxel belongs to the *g*-th partition and ℐ_*g*,*j*_ = 0 otherwise. The parameters λ_*S*_, λ_*G*_ ≥ 0 adjust the balance between model fitting precision (first term in Eq 3), voxel-wise sparseness (second term), and group-wise sparseness (third term). λ_*S*_ > 0 and λ_*G*_ > 0 imposes both voxel-wise and group-wise sparseness. We refer to this case as sparse group LASSO (sgLASSO). For λ_*S*_ > 0 and λ_*G*_ = 0 the above therefore yields the standard sparse LASSO (sLASSO) while λ_*S*_ = 0 and λ_*G*_ > 0 defines the group LASSO (gLASSO).

The advantage of group LASSO is that prior knowledge of possibly related input features can be incorporated by suitably setting the indicator function ℐ_*g*,*j*_. We exploit this advantage to incorporate functional localization within brain regions. Input to the algorithm consists of a feature matrix formed from standardised brain activation Z-scores **x**_*i*_ of 62 subjects, where 31 rows represent control subjects (label *y*_*i*_ = −1) and 31 rows depressive patients (label *y*_*i*_ = +1). 105 brain areas were considered in the analysis, i.e., *G* = 105 and ℐ_*g*,*j*_ = 1 if the *j*-th voxel belongs to the *g*-th brain area.

Once the weight vector **w** is determined, the logistic regression model can be used as a discriminant function for the binary classification (in the fashion of the most probable explanation, a special case of the maximum a-posteriori probability). For each feature vector **x**, the label *y* is determined as follows:

$$\begin{array}{c} y=\{\begin{array}{cc} & +1\;\text{if}\;P(y=+1|x)>P(y=-1|x)\text{,}\;\text{i.e.,}\;x^{T}w>0\\ & -1\;\text{otherwise,}\;\text{i.e.,}\;x^{T}w<0 \end{array} \end{array}$$

Unless specified otherwise, we evaluated classification performance in our study based on this discriminant function.

#### Support Vector Machine

The SVM training algorithm is a non-probabilistic binary classifier that represents samples as points in space, so that samples of different categories are separated by a margin as large as possible (large margin principle of separation [13]). Here we use parameter-free linear (hard margin) SVM [12].

#### Random Forest

The Random Forest algorithm is an ensemble learning method that constructs a multitude of decision trees and yields the mode of the class output by individual trees as result [25].

### Performance Criteria

Parameter tuning of LASSO algorithms was based on the mean log likelihood *μ*_log* L*_, a standard performance measure for probabilistic models:

$$\begin{array}{c} \mu_{\log L}=\frac{1}{n}\sum_{i=1}^{n}\log P\left(y_{i}|x_{i};w\right). \end{array}$$ (4)

For better visualization of the class probability distributions, we calculated the logarithmic odds ratio $\log(\frac{P(y=+1|\text{x})}{P(y=-1|\text{x})})$ and normalized it by the maximum logarithmic odds ratio of all test data. In the following, we refer to these values as *normalized discriminative scores*.

For performance comparison of probabilistic LASSO models with non-probabilistic models such as SVM, we used the four criteria defined below. We defined patients who were correctly classified as depressive, as true positives (TP), and those incorrectly classified as healthy, as false negatives (FN). We referred to control subjects who were correctly classified as healthy, as true negatives (TN), and those incorrectly classified as depressed, as false positives (FP).

*Sensitivity* and *Specificity* evaluate the probability of correct diagnosis of patients and control subjects, respectively:

$$\begin{array}{ccc} Sensitivity & = & \frac{\#\text{correctly}\;\text{classified}\;\text{patients}}{\#\text{all}\;\text{patients}}\\ & = & \frac{\#TP}{\#TP+\#FN} \end{array}$$ (5)

$$\begin{array}{ccc} Specificity & = & \frac{\#\text{correctly}\;\text{classified}\;\text{controls}}{\#\text{all}\;\text{controls}}\\ & = & \frac{\#TN}{\#TN+\#FP} \end{array}$$ (6)

*Accuracy* and *Fscore* evaluate overall performance. The *Fscore* provides a more rigorous measure when the number of control subjects is larger than that of patients, in which case, true negatives can dominate the *Accuracy* measure.

$$\begin{array}{ccc} Accuracy & = & \frac{\#\text{correctly}\;\text{classified}\;\text{subjects}}{\#\text{all}\;\text{subjects}}\\ & = & \frac{\#TP+\#TN}{\#TP+\#FN+\#TN+\#FP} \end{array}$$ (7)

$$\begin{array}{ccc} Fscore & = & \frac{2}{1/Precision+1/Recall}\\ & = & \frac{\#TP}{\#TP+(\#FN+\#FP)/2} \end{array}$$ (8)

where $Precision=\frac{\#TP}{\#TP+\#FP}$, is the probability that a subject classified as a patient really is a patient and $Recall=\frac{\#TP}{\#TP+\#FN}$ (= *Sensitivity*), the probability that a patient is classified as a patient.

### Parameter Tuning and Performance Evaluation

Classification performance of the algorithms was evaluated in a 10-fold cross-validation. In order to account for model variability, the procedure was repeated 100 times, each time pseudo-randomly shuffling sample contributions to the training and test datasets.

**Fig 2:**
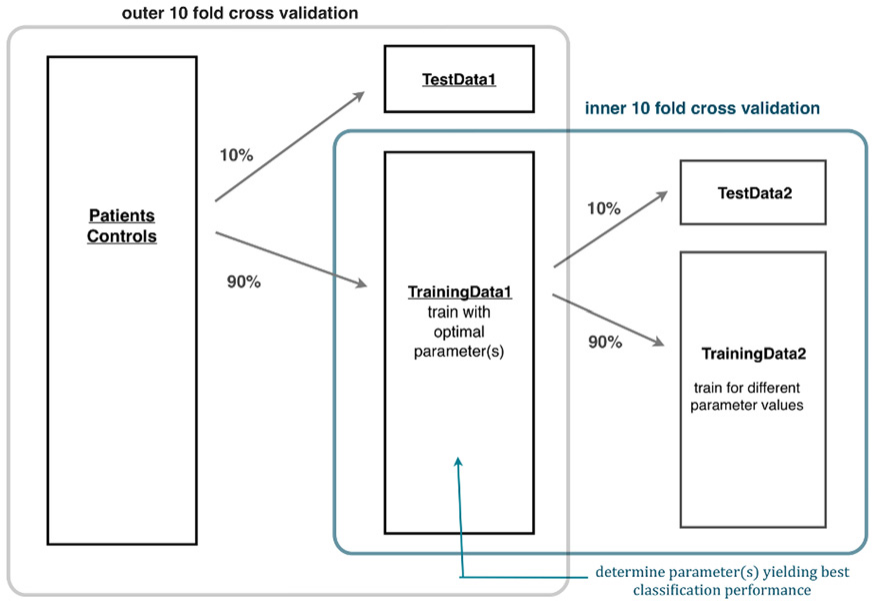
Nested 10-fold cross-validation. 10% of the data were defined as test dataset (TestData1) while the remaining 90% (TrainingData1) were used to evaluate the best performing regularization parameter λ_*opt*_ (= λ_*Sopt*_ in the case of sLASSO, = λ_*Gopt*_ in the case of gLASSO, = (λ_*Sopt*_, λ_*Gopt*_) in the case of sgLASSO) in a 10-fold cross-validation (inner 10-fold cross-validation). Parameters thus determined were then used to train TrainingData1 and to evaluate the final performance using TestData1. The same procedure was repeated ten times, altering the set of train and test data each time, so as to complete a 10-fold cross-validation (outer 10 fold cross-validation).

For SVM and Random Forest, conventional 10-fold cross-validation was used. For LASSO algorithms, cross-validation was conducted in a nested manner as described below, in order to account for parameter validation (Fig 2).

#### Nested 10-fold cross-validation

The first cross-validation (inner cross-validation) served to optimize regularization parameters, while the second cross-validation (outer cross-validation) evaluated predictive performance when using (optimal) parameters obtained in the first cross-validation to a new dataset. For sLASSO 200 logarithmically distributed values of λ_*S*_ in the range of [0.01 0.2512] were evaluated, while for gLASSO we chose 100 logarithmically distributed values of λ_*G*_ in the range of [0.1 0.6310]. For sgLASSO we used 200 combinations of 20 logarithmically distributed values of λ_*S*_ and 10 logarithmically distributed values of λ_*G*_ in the range of [10^−6^ 0.1] and [0.0631 0.3162], respectively. The approximate range of the parameters was determined by grid search.

In detail, the procedure consisted of following twelve steps:

1. Data from controls and patients were divided into 10 non-overlapping sets with approximately equal number of subjects.
2. One of these datasets was taken as test data (TestData1) and the rest were used as training data (TrainingData1).
3. Step 1 was repeated for TrainingData1, i.e., it was divided into 10 separate and non-overlapping sets.
4. One of the datasets produced in Step 3 was used as test data (TestData2) and the rest were used as training data (TrainingData2).
5. Models were trained for a range of parameters using TrainingData2.
6. Class probabilities for TestData2 were predicted using the resulting models.
7. Steps 4 to 6 were repeated ten times, each time defining different datasets as TestData2 and TrainingData2.
8. The mean log likelihood (*μ*_log*L*_) was evaluated for each setting of the parameters and test dataset and the optimal parameter (λ_*Sopt*_, λ_*Gopt*_ and λ_*opt*_ = (λ_*Sopt*_, λ_*Gopt*_) for sLASSO, gLASSO and sgLASSO respectively), for which *μ*_log*L*_ is maximized was determined.
9. A model was estimated using TrainingData1 and λ_*max*_.
10. Class probabilities for TestData1 were predicted.
11. Steps 2 to 9 were repeated ten times, each time choosing a different dataset from the 10 sets produced in Step1, as TestData1.
12. Prediction performance was evaluated.

Fig 2 illustrates the procedure. As for SVM and Random Forest, this procedure was repeated 100 times, each time pseudo-randomly shuffling the combination of data in Step1.

All routines were programmed in Matlab (Mathworks). For SVM and Random Forest, functions provided in the Matlab optimization Toolbox were used while for LASSO algorithms we made use of the sparse learning package (SLEP) provided by Liu et al. [29].

## Results

### Task Performance and SPM Evaluation

#### Semantic Verbal Fluency

The number of successfully completed tasks was significantly higher for controls than for depression patients (two sample t-test, *p* = 0.03). The same was true for corresponding button press reaction times (*p* = 0.0098). On average, the control group accomplished 29.19 trials out of 30 correctly with a standard deviation of 1.51, while the depression group completed on average 27.81 ± 3.12 trials. Reaction times for the control group averaged 1.28 ± 0.32 seconds, while the depression group mean was 1.50 ± 0.33 seconds.

A group-wise two-sample t-test of SPM-evaluated brain activation revealed no significant difference in brain activation between depressed and control subjects (uncorrected *p* = 0.01).

#### Phonological Verbal Fluency

As in the semantic task, a two-sample t-test revealed that phonological task performance was significantly different between the two subject groups. The number of successfully completed tasks totaled 29.19 ± 1.52 and 26.58 ± 2.86 for control and depression group, respectively (*p* = 0.0003). Reaction times for the control group averaged 1.39±0.36 seconds while for the depression group it was 1.65 ± 0.34 seconds (*p* = 0.0047) (Table 1)

**Table 1:** Study participants and behavioral performance (mean±std).

	Control group	Depression group	*p*
n	31	31	
age	33.45 ± 12	38.81 ± 9.76	0.06
gender (male)	15	16	0.80
reading test (JART)	113.33 ± 8.51	109.29 ± 10.19	0.10
**Semantic task:**			
successful trials	29.19 ± 1.51	27.81 ± 3.12	0.03
mean reaction time (s)	1.28 ± 0.32	1.50 ± 0.33	0.0098
**Phonological task:**			
successful trials	29.19 ± 1.54	26.58 ± 2.86	0.0003
mean reaction time (s)	1.39 ± 0.36	1.65 ± 0.34	0.0047

SPM group-wise analysis for the phonological task revealed 12 voxel clusters that were significantly more activated in controls than depressive subjects (uncorrected *p* = 0.01). They were mainly located around the *bilateral lingual* and *cerebellar cortex*, as well as the *bilateral cuneus* and *precuneus*.

### Parameter Dependence

**Fig 3:**
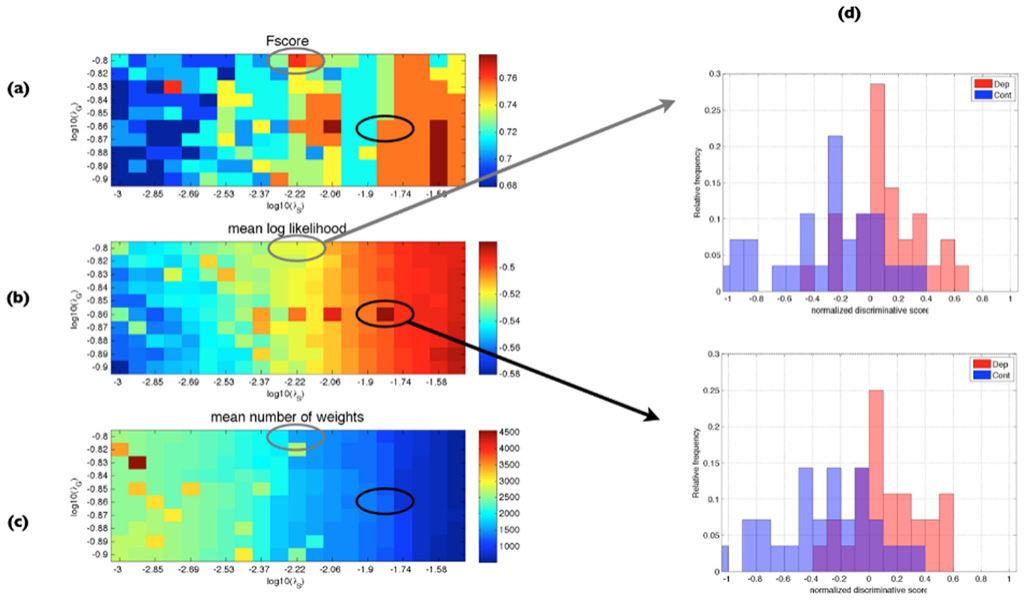
Maximum mean log likelihood provides better separation of normalized discriminative score distributions than the Fscore. (a) *Fscore*, (b) mean log likelihood *μ*_log *L*_, and (c) the number of non-zero weights for different combinations of the regularization parameters λ_*S*_ and λ_*G*_, in an inner cross-validation (see Material and Methods) of sgLASSO applied to the semantic data. (d) Distribution of discriminative scores (normalized log odds ratios) at the maximum mean log likelihood (indicated by the black circle in (b)). (e) Distribution of discriminative scores at a non-optimal parameter setting (indicated by the grey circle in (b)). For this case, the *Fscore* (a, grey circle) is higher, but the separation of discriminative score distributions (d, top) is less clear than at the optimal setting (a, black circle, d, bottom).

As the performance of LASSO algorithms depend on regularization parameters (λ_*S*_ for sLASSO, λ_*G*_ for gLASSO and λ = (λ_*S*_, λ_*G*_) for sgLASSO, respectively), we first verified the regularization parameters evaluated as optimal in a grid search combined with a 10-fold cross validation (see Materials and Methods for details). We compared *Fscores*, mean log likelihood and the number of selected weights of the test data yielded when using optimal parameters and when using other specific values of the regularization parameters (Fig 3a–3c). Naturally, with larger settings of λ_*S*_ and λ_*G*_, the number of non-zero weights decreased (Fig 3c). Plotting of respective discriminative score distributions (Fig 3d) confirmed that distributions obtained with the parameters yielding a higher mean log likelihood were favorable to those yielding a lower mean log likelihood, even if the *Fscore* was the same (Fig 3a–3c). This validated our decision to base the choice of optimal parameters on the highest mean log likelihood.

**Fig 4:**
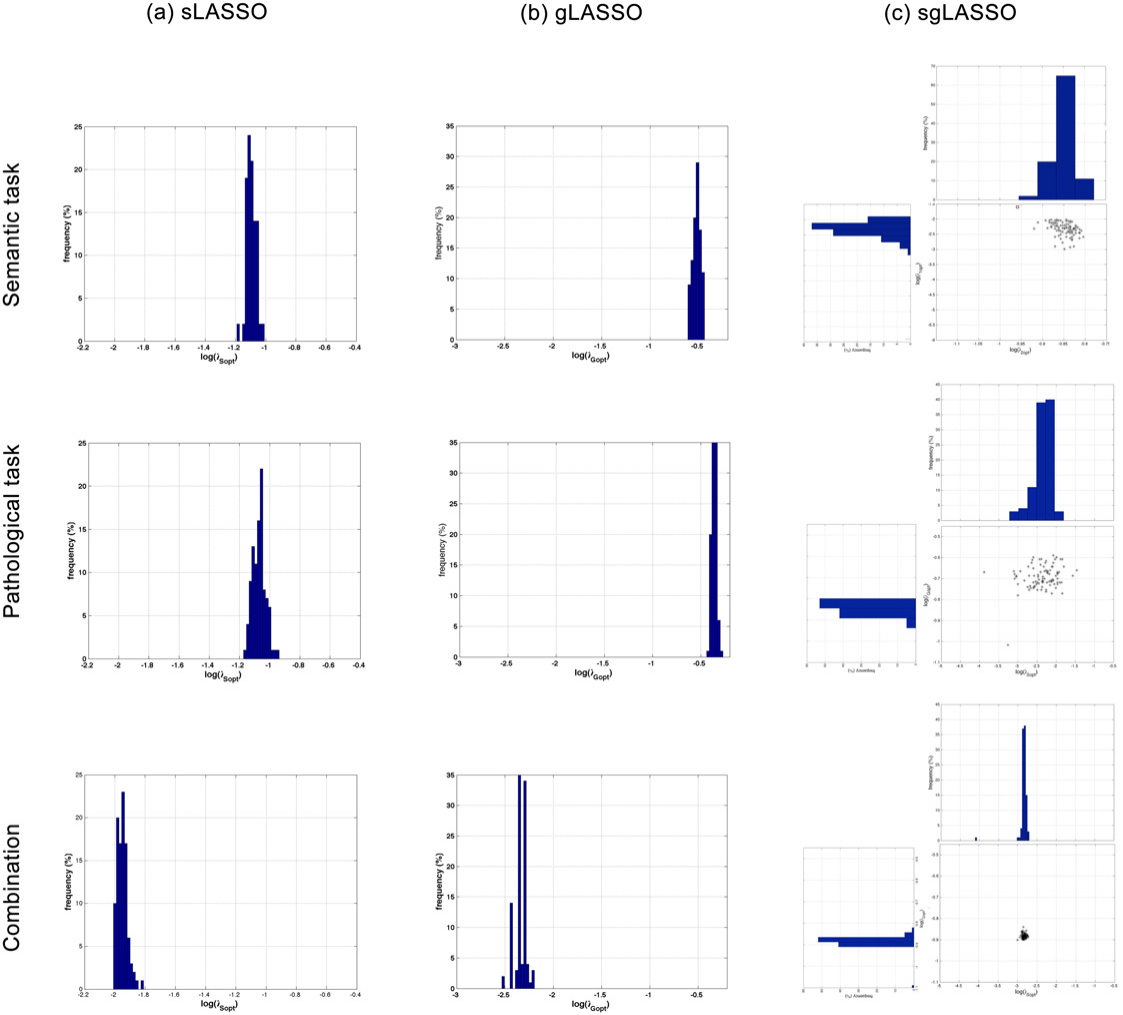
Histograms confirm stability of the parameters yielding the best classification performance in each cross-validation. For all datasets and algorithms, the distribution of optimal parameters over 100 nested 10-fold cross-validations shows a roughly Gaussian distribution.

As a second step, we verified by visual means (Fig 4) that the optimal parameters chosen in each cross validation were not randomly distributed. In standard LASSO (Fig 4a), the optimal parameter λ_*Sopt*_ was similar for both semantic and phonological task data, while it was smaller for the combined dataset. A similar result was found for the distribution of λ_*Gopt*_ of the group LASSO (Fig 4b). In the two-dimensional parameter space of sgLASSO, λ_*Sopt*_ and λ_*Gopt*_ had unimodal distributions (Fig 4c).

### Classification Performance

**Fig 5:**
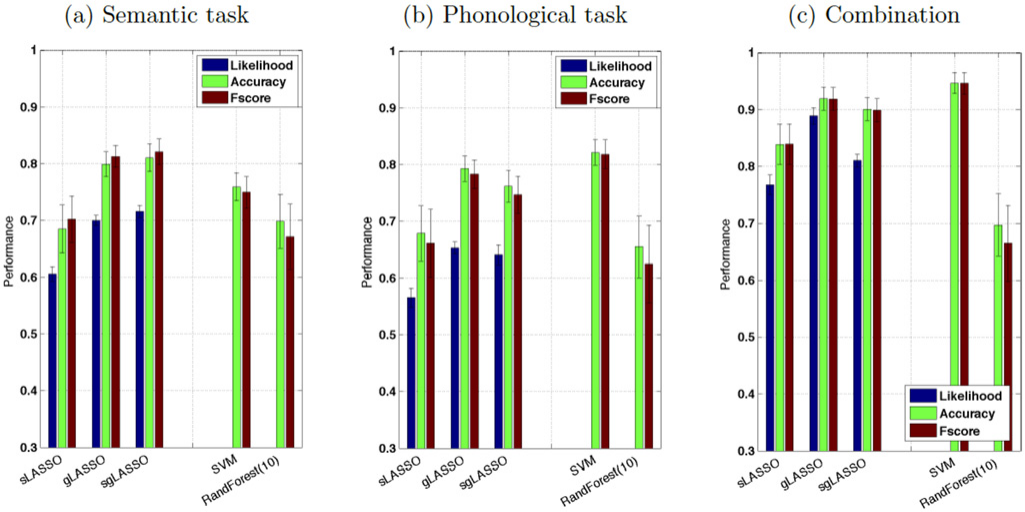
Relative performance of gLASSO, sgLASSO and SVM, depended on the dataset, while sLASSO and Random Forest were generally outperformed by the other algorithms. (a) semantic verbal fluency, (b) phonological verbal fluency and (c) combined datasets. Classification performance was significantly different between all algorithms and significantly higher for each algorithm with the combined dataset (*p* < 0.001, u-test).

Fig 5 and Table 2 summarize the classification results:

**Table 2:** Classification Performance (mean±std over 100 10-fold cross-validations).

Data & Algorithm	Sensitivity	Specificity	Accuracy	*Fscore*	Likelihood
**Semantic task**					
sLASSO	74.52±5.59	62.45±5.64	68.48±4.22	0.66±0.04	0.61±0.01
gLASSO	87.16±2.79	72.71±3.98	81.29±1.95	0.70±0.01	0.70±0.01
sgLASSO	86.74±3.04	75.39±3.69	81.06±2.41	0.82±0.02	0.72±0.01
SVM	72.23±3.91	79.61±3.11	75.92±2.45	0.75±0.03	-
Random Forest	62.06±7.25	77.55±6.37	69.81±4.75	0.67±0.06	-
**Phonological task**					
sLASSO	63.16±7.66	72.55±5.53	67.85±4.89	0.66±0.06	0.57±0.02
gLASSO	74.26±3.84	83.71±2.57	78.98±2.47	0.78±0.03	0.65±0.01
sgLASSO	70.58±4.48	81.77±3.05	76.18±2.74	0.74±0.032	0.64±0.02
SVM	80.74±3.96	83.52±1.44	82.13±2.22	0.82±0.03	-
Random Forest	58.10±8.98.37	57.00±8.19	65.50±5.41	0.62±0.07	-
**Combination**					
sLASSO	84.61±4.60	83.03±4.80	83.82±3.57	0.84±0.04	0.77±0.02
gLASSO	91.35±2.90	92.55±2.70	91.95±2.02	0.92±0.02	0.89±0.02
sgLASSO	88.45±2.72	91.71±3.12	90.08±2.02	0.90±0.02	0.90±0.02
SVM	94.48±2.48	94.87±2.43	94.68±1.86	0.95±0.02	-
Random Forest	60.65±8.34	78.74±5.93	69.69±5.42	0.66±0.07	-

#### Semantic Verbal Fluency

Using sLASSO, depressed and control subjects could be classified with 68.48 ± 2.47% accuracy (sensitivity 74.52 ± 5.59%, specificity 62.45 ± 5.64%), *Fscore* = 0.70 ± 0.04 and mean likelihood of *L* = 0.61 ± 0.01 (see Fig 5 and Table 2. Standard deviations were calculated from 100 evaluations of the models obtained in a nested 10-fold, cross-validation repeated 100 times with shuffled training data).

gLASSO and sgLASSO performed significantly better than sLASSO (*p* < 0.001, u-test). gLASSO yielded 81.29 ± 1.95% accuracy (87.16 ± 2.79% sensitivity and 72.71 ± 3.98% specificity) and *Fscore* = 0.70 ± 0.01 with a mean likelihood of *L* = 0.70 ± 0.01, while sgLASSO yielded 81.06 ± 2.41% accuracy (86.74 ± 3.04% sensitivity and 75.39 ± 3.69% specificity) and *Fscore* = 0.82 ± 0.02 with a mean likelihood of *L* = 0.72 ± 0.01. Classification performances of SVM and Random Forest were significantly lower than those of sLASSO, gLASSO, and sgLASSO (*p* < 0.001, u-test).

#### Phonological Verbal Fluency

Here, sLASSO yielded an accuracy of 67.85 ± 4.89% (sensitivity 63.16 ± 7.66%, specificity 72.55 ± 5.53%) and *Fscore* = 0.66 ± 0.06 with a mean likelihood of *L* = 0.57 ± 0.02. Again, gLASSO and sgLASSO outperformed sLASSO (p < 0.001, u-test). Performance values for gLASSO were: accuracy 78.98 ± 2.47 (sensitivity 74.26 ± 3.84%, specificity 83.71 ± 2.57%), *Fscore* = 0.78 ± 0.03 and *L* = 0.65 ± 0.01. Those for sgLASSO were: accuracy = 76.18 ± 2.74% (sensitivity 70.58 ± 4.48%, specificity 81.77 ± 3.05%), *Fscore* = 0.74 ± 0.032 and *L* = 0.64 ± 0.02.

For this dataset, performance of SVM achieved an accuracy of 82.13 ± 2.22% and *Fscore* of 0.82 ± 0.03, which was significantly higher than performances of gLASSO and sgLASSO (*p* < 0.001, u-test).

#### Combination of Semantic and Phonological Verbal Fluency Data

Classification performance of all three LASSO algorithms, as well as SVM, significantly improved when data from the semantic and phonological tasks (*p* < 0.001, u-test) were combined by simple vector concatenation. For sLASSO, the performance improved to: 83.82 ± 3.57% accuracy (sensitivity 84.61 ± 4.60% and specificity 83.03 ± 4.80%), *Fscore* = 0.84 ± 0.04 and *L* = 0.77 ± 0.02.

For gLASSO and sgLASSO, classification performance was even higher, showing following performance: accuracy 91.95 ± 2.02% (sensitivity 91.35 ± 2.90% and specificity 92.55 ± 2.70%), *Fscore* = 0.92 ± 0.02 and *L* = 0.89 ± 0.02, for gLASSO and accuracy 90.08 ± 2.02% (sensitivity 88.45 ± 2.72%, specificity 91.71 ± 3.12%), *Fscore* = 0.90 ± 0.02 and *L* = 0.89 ± 0.02 for sgLASSO.

SVM achieved a classification accuracy of 94.68 ± 1.86% and an *Fscore* of 0.95 ± 0.22, while Random Forest performed significantly more poorly than all other methods (*p* < 0.001, u-test).

### Discriminative Scores

**Fig 6:**
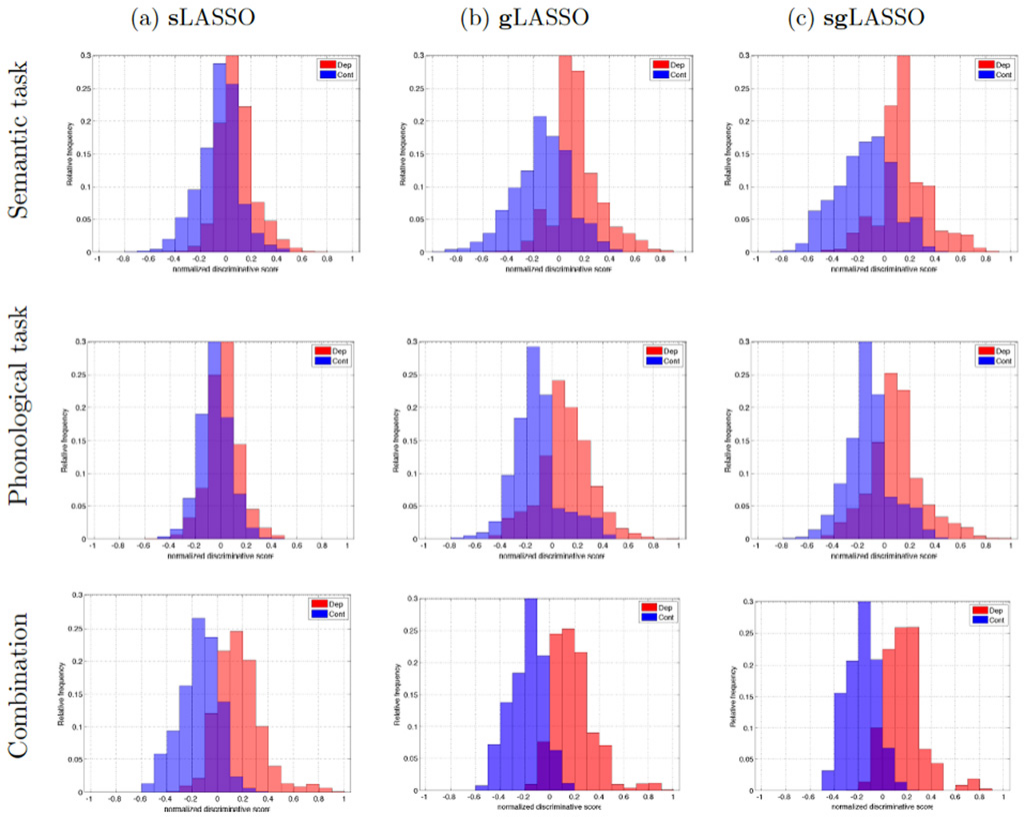
For all algorithms the separation of class probability distributions became clearer when semantic and phonological data were combined. normalized discriminative score distribution was evaluated over all 1000 test datasets produced during the nested cross validation. The discriminative score is given by the logarithmic odds ratio and is here normalized by the maximum value of all test data.

Normalized Discriminative Score distributions for patients and control subjects largely overlap when sLASSO was applied to semantic or phonological task data (Fig 6a). In comparison, distributions resulting from gLASSO and sgLASSO, are well separated with little overlap.

For all three LASSO algorithms, the separation becomes clearer when the two datasets are analyzed in a combined fashion. Especially with gLASSO and sgLASSO, the discriminative score showed good separation of patients and control subjects, with false positives and false negative located only near the classification border. All false positive subjects showed a discriminative score < 0.2 and all false negative subjects had scores > −0.2.

For all three datasets, false negative and false positive subjects in each cross-validation were confirmed to coincide. All false negative subjects in the classification of semantic and phonological data showed only weak symptoms of depression six weeks after participation in our study and medical treatment with Hamilton Rating Scale of Depression scale score (*HRSD*) smaller than 11. We refer to these patients as *in remission*. However, only for some, the *HRSD* decreased more than 50% of the original score (response). For all patients misclassified based on the combined dataset, remission as well as response could be confirmed. For misclassified control subjects (false positives), no common characteristic could be found. However, only two control subjects were misclassified. One of them had a history of a manic episodes and the other showed very low performance in the phonological task. The true relation of misclassification and biomarkers has to be confirmed with larger datasets.

### Contributing Features and Brain Areas

**Fig 7:**
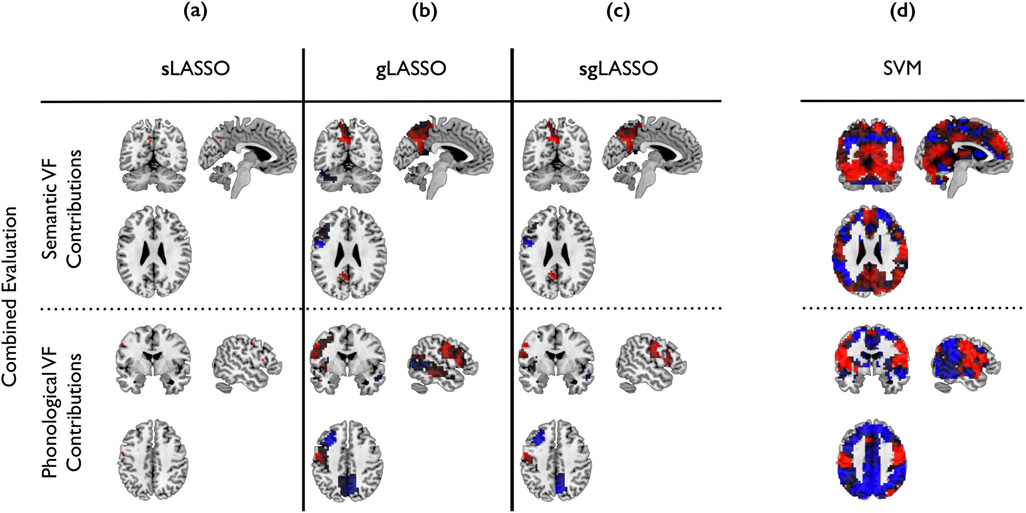
gLASSO constrains the number of groups of voxels (example combined dataset). This avoids the problem seen with sLASSO, where single voxels may erroneously suggest that brain areas containing those voxels distinguish between depressed and non-depressed patients. (a) For sLASSO, the number of non-zero weights is naturally sparse. Contributing voxels tend to be scattered, thus jeopardizing predictions in the case of even slight data distortion. (b) In gLASSO this is prevented by assigning voxels to groups. (c) sgLASSO further sparsifies contributing voxels within these groups, thus eliminating redundant weights, but preserving topological continuity. (d) The SVM algorithm uses all voxels to estimate the classification model (Plotted with mricron [30]). Positive weights are displayed in red, indicating voxels typically more highly activated in depressed subjects than in control subjects. Negative weights are displayed in blue, indicating voxels typically more highly activated in control subjects. Only voxels selected in more than 80% of the 1000 models evaluated in the hundred 10-fold nested cross-validation are displayed. Contributions in the evaluation of the separated datasets were similar to that of the combined evaluation, but less stable with respect to selection frequency in the cross validation models (see Fig 8).

In our LASSO approach, features relevant to classification can easily be identified. We only need to look at voxels with non-zero weights. In Fig 7 we illustrate the effect for the weights selected when the algorithms are applied to the combined verbal fluency dataset. Positive weights (in red) indicate voxels that are typically more highly activated in depressed subjects than in control subjects. Conversely, negative weights (in blue) indicate voxels that are typically more highly activated in control subjects than in depressed subjects. The number of selected voxels varies depending on the algorithm employed (Table 3). While contributing voxels in sLASSO (Fig 7a) were naturally very sparse and often isolated, group-wise sparsification with gLASSO (Fig 7b) resulted in a large number of non-zero weights. The sum of positive and negative voxels covered entire brain areas while with sgLASSO (Fig 7c), voxels within these brain areas were sparsified as well, but still spatially connected. As anticipated, this allows for isolation of relevant regions in the brain, rather than single voxels. SVM (Fig 7d) attributes weights to all voxels, making it difficult to identify brain areas most relevant to the classification.

**Table 3:** Feature Selection (mean±std over 100 10-fold cross-validations, i.e. 1000 classification models).

Data & Algorithm	#selected voxels in each model	#voxels (#brain areas) selected at least once	max occurrence of same voxel	#voxels with > 80% occurrence
**Semantic task**				
sLASSO	19.14 ± 5.91	450 (71)	89.9%	1
gLASSO	2497.95 ± 527.50	6906 (31)	100%	1703
sgLASSO	1956.87 ± 699.16	9041 (47)	100%	727
SVM	14055 (all)	14055 (all)	100%	14055 (all)
**Phonological task**				
sLASSO	20.14 ± 6.70	478 (65)	92.8%	1
gLASSO	2438.32 ± 597.45	5931 (26)	100%	1885
sgLASSO	1838.72 ± 1227.52	8852 (43)	100%	395
SVM	14055 (all)	14055 (all)	100%	14055 (all)
**Combination**				
sLASSO	41.98 ± 5.41	735 (54+58)	98.60%	6
gLASSO	6094.51 ± 1001.26	13241 (26+32)	100%	3926
sgLASSO	3387.33 ± 750.13	10695 (19+28)	100%	1878
SVM	14055 (all)	14055 (all)	100%	14055 (all)

#### Semantic Verbal Fluency

**Fig 8:**
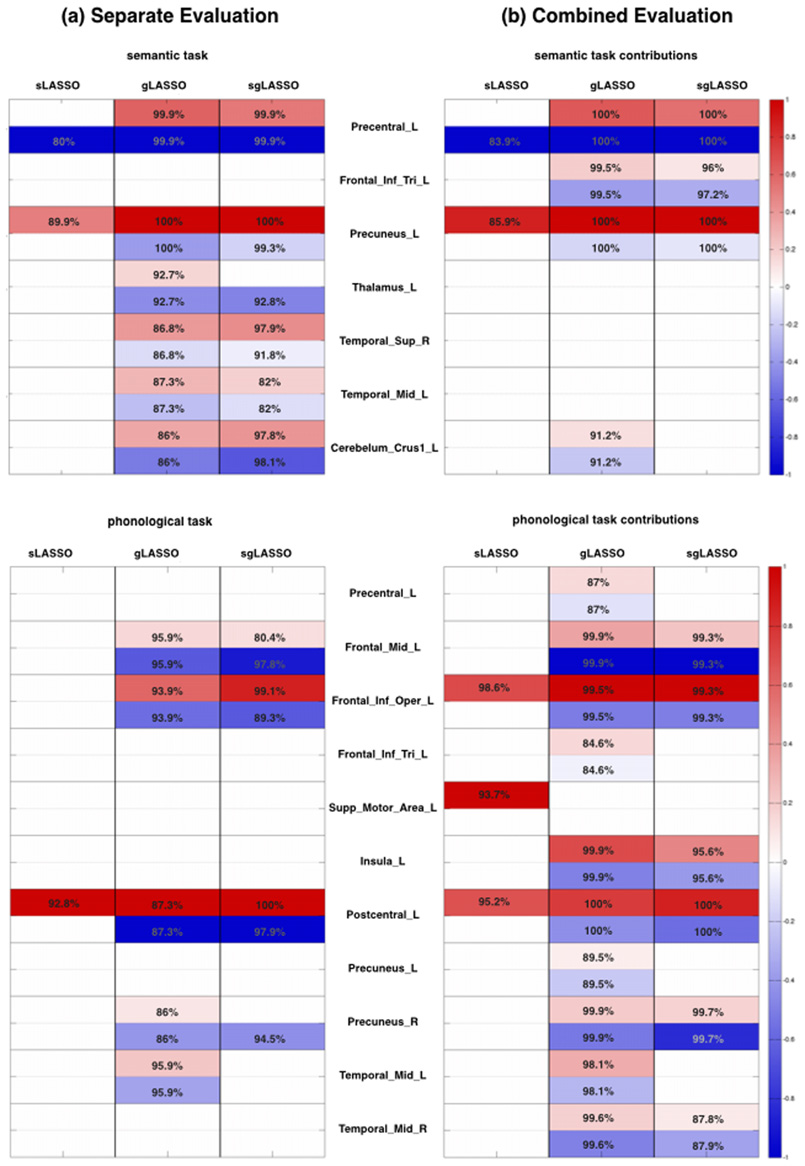
In the combined evaluation, model stability is higher than in the separate evaluation. This is indicated by the selection frequency of the same brain areas during the cross validation. The frequency is generally lower for (a) separate evaluation than in the (b) combined evaluation. Only brain areas selected in more than 80% of all cross validated models are given. Numbers indicate the selection frequency. The colors indicate average values over all positive and negative weights (normalized by the highest average positive and negative value over all brain areas, resp.). Areas more highly activated in control subjects than in depressed subjects are in blue; areas more highly activated in depressed subjects than in control subjects are in red. For gLASSO, the sum of negatively and positively weighted voxels cover 100% of the brain area of interest, so that the selection frequency is the same.

Using sLASSO, on average 19.14 ± 5.91 voxels were selected in individual models of cross-validations (Table 3). However, the sum of all voxels selected in all evaluated models together, resulted in 450 different voxels distributed over 71 brain areas. Voxels of only two brain areas contributed to the classification in more than 80% of the models, namely *left precuneus*, which also yielded the highest mean average positive weight and *left precentral cortex*, which held the highest average negative weight (Fig 8(a), Semantic task).

As desired, gLASSO limits the number of voxels based on whole brain areas. Accordingly, the average number of voxels selected in individual models was larger than that selected with sLASSO: 2497.95 ± 527.50 (Table 3). Altogether 6,906 different voxels originating from 31 brain areas were selected. The following brain areas were selected in more than 80% of all models: *left precuneus*, *left precentral cortex*, *right superior temporal cortex*, *left cerebellum (Crus1)*, *left middle temporal cortex* and *left thalamus*. These areas were assigned the highest average negative and the highest average positive weights. Hereby, the *left precuneus* contributed to all classification models (Fig 7(a), Semantic task), i.e., 100% of the time.

Results from sgLASSO were similar to those of gLASSO. Since an additional parameter (λ_*S*_) sparsifies the number of contributing voxels in each brain area, on average 1956.87 ± 699.16 weights remained in each estimated model (Table 3). During cross-validation, altogether 9,041 voxels originating from 47 brain areas were selected. Brain areas selected more than 80% of the time coincided with those of gLASSO. However, the *left thalamus* contributed negative weights only, while other brain areas contained positive as well as negative weights. Again, the *left precuneus* was selected in all classification models.

#### Phonological Verbal Fluency

sLASSO resulted in 20.14 ± 6.70 remaining voxels in classification models, summing to 478 voxels from 65 different brain areas. Only voxels located in the *left postcentral gyrus* contributed to more than 80% of all cross validated models (Table 3). These voxels had only positive weights (Fig 8(a), Phonological task).

gLASSO selected 5,931 different voxels covering 26 brain areas. On average 2438.32 ± 597.45 voxels remained in individual models (Table 3). Brain areas that contributed most frequently with the highest average positive weights coincided with those having the highest average negative weights. As above, these areas were selected in more than 80% of all models: *left postcentral gyrus*, *left inferior frontal operculum*, *left middle temporal cortex*, *left middle frontal cortex* and *right precuneus*.

On average, 1838.72 ± 1227.52 voxels were selected when applying sgLASSO, the union of which amounted to 8,852 different voxels from 43 different brain areas (Table 3). Four brain areas were selected in more than 80% of the models. The following brain areas had the highest average positive as well as highest average negative weights: *left post central gyrus*, *left inferior frontal operculum* and *left middle frontal cortex*. The positive weights of the *left post central gyrus* were selected in all models. The *right precuneus* showed positive as well as negative weights; however, positive weights appeared in only 70.1% of the models and were therefore disregarded. Negative weights, in contrast, contributed to the classification model 94.50% of the time. While the *left postcentral gyrus* was selected only 92.8% of the time with sLASSO, it contributed in 100% of the models when using gLASSO and sgLASSO (Fig 8a).

#### Combined Verbal Fluency Data

When the two verbal fluency datasets were combined, brain areas contributing to the classification models are similar, but became more distinct with respect to the relative frequency of contribution to the cross validated models. For all three LASSO algorithms, the selection frequency of the brain areas is generally higher than in the analysis of the individual dataset (Fig 8), indicating higher model stability.

Using sLASSO, each model contained on average 41.98 ± 5.41 voxels, where on average 20.30 ± 3.70 voxels came from semantic data and 21.68 ± 4.11 from phonological data (Table 3). Brain areas most frequently contributing to the semantic data were *left precuneus* and *left precentral cortex* with positive and negative weights, respectively. Positive weights in the phonological data were located in the *left supplementary motor area (SMA)*, *left inferior frontal operculum* and *left post central cortex*, while no negative weights could be found. Voxels attributed non-zero weights in semantic data were in similar locations to those attributed non-zero weights when analyzing semantic data alone. In contrast, *left inferior frontal operculum* and *left supplementary motor area (SMA)* contained non-zero weights when processing phonological data alone, but were not selected when semantic and phonological data were analyzed together (Fig 8(a) and 8(b), Phonological task).

For gLASSO, the number of selected voxels amounted to 6094.51 ± 1001.26 of which 2681.98 ± 715.75 were selected from the semantic dataset and 3412.53 ± 403.83 from the phonological dataset (Table 3). Brain activation of four brain areas exhibited during the semantic task, was discriminative in more than 80% of all models: *left precuneus, left precentral gyrus*, *left inferior frontal cortex (pars triangularis)* and *left cerebellum (crus1)*. All of them contained positive as well as negative weights. The *left inferior frontal cortex (pars triangularis)* was not prominent when classifying semantic data alone. On the other hand *left thalamus* and the *temporal* areas did not frequently contribute to models resulting from the combined data.

Phonological data contributed to the classification with weights in eleven different brain areas, namely *left inferior frontal operculum*, *left post central gyrus*, *left insula*, *left middle frontal cortex*, *left middle temporal cortex*, *right precuneus*, *right middle temporal cortex*, *left inferior frontal cortex (pars triangularis)*, *left precentral gyrus and left precuneus*. As in the case of semantic data, all of these areas contained negative as well as positive values. Here, the number of contributing brain areas in more that 80% of all models has increased.

As expected from the sparseness constraint on the voxels, fewer non-zero weight voxels were observed in models trained with sgLASSO than with gLASSO (3387.33 ± 750.13 with 1185.28 ± 409.55 contributed by semantic data and 2202.05 ± 454.76 by phonological). Areas that contributed most frequently to classification were *left precuneus*, *left precentral cortex*, *left inferior frontal cortex (pars triangularis)* with the semantic data and *left inferior frontal operculum*, *left postcentral gyrus*, *left insula*, *left middle frontal cortex*, *right precuneus*, *right middle temporal cortex* with the phonological data. All areas contained negative as well as positive weights.

Using gLASSO and sgLASSO, positive as well as negative weights of *left precentral cortex* and *left precuneus* (semantic data) and *right post central cortex* (phonological data) contributed to classification models 100% of the time.

## Discussion

We applied three variants of logistic regression LASSO and two deterministic classification algorithms, SVM and Random Forest, to fMRI data from semantic and phonological verbal fluency tasks to demonstrate the advantages of group LASSO for classification of fMRI data, specifically the ability to identify relevant brain regions of interest.

More than 90% accuracy was achieved in distinguishing depression patients from matched healthy control subjects when applying gLASSO, sgLASSO, and SVM to the combination of the two datasets. Our results surpass classification performance in similar studies involving depression related fMRI and SVM (65% to 86% [9, 10, 14]). Similarly accurate prediction results with respect to depression-related MRI data, to the best of our knowledge have only been achieved by Mwangi et al. [31]. However, in contrast to our study, the latter authors relied on structural MRI data from patients considered to be chronically depressed. For such patients, structural brain changes are very likely [32]. Depression studies based on whole structural brain data are the most common and are reviewed in [33]. Except for the study mentioned above [31], the maximum classification accuracy between healthy controls and depression patients in other relevant studies was 70%. Considering that in general, classification accuracy for treatment responsive and non-responsive subjects is generally 80% [33], we assume that the classification success of [31] results from the homogenous, chronically depressed patient group. Kipli et al. also provide a comparative study on classification accuracy for various brain volume attributes and varying combinations of machine learning algorithms for feature selection and classification [34]. Here, the maximum classification accuracy for depression reached 85.3%. However, the evaluation of the algorithms was solely based on classification accuracy for preselected brain volumetric attributes. Sparse algorithms were not considered.

### sLASSO vs gLASSO/sgLASSO

Since sLASSO prediction relies on mostly spatially isolated voxels (Fig 8a), activities shifted to adjacent voxels due to inter-subject variability can lead to classification errors. In contrast, gLASSO and sgLASSO estimate class label probability from values of spatially continuous voxels (Fig 8b and 8c). These voxels not only indicate brain regions of interest, thereby facilitating interpretation of results; brain activity shifted due to inter-subject variability is likely to be retrieved by neighboring weights. This argument is supported by the small number of commonly selected voxels over different runs of the cross-validation (Table 3).

In the application of sLASSO to semantic and phonological task data, the number of voxels selected in at least one of the evaluated models was 450 and 478, selected from 65 and 71 brain areas, respectively. Only a single voxel was selected commonly in more than 80% of the models in the cross-validation. In contrast, the highest occurrence rate in gLASSO and sgLASSO reached 100%. More than 500 voxels were selected in more than 80% of the models for each of the algorithms and datasets. These numbers increased when the two datasets were combined (Table 3).

### gLASSO vs sgLASSO

The relative performance of gLASSO and sgLASSO varied depending on the datasets. Both algorithms attributed the highest weights to the same brain areas and even the same locations within these brain areas (Fig 8b and 8c). While sgLASSO can be more effective in selecting truly relevant voxels, a more relaxed voxel selection with gLASSO might be advantageous when taking variability in individual brain areas and activation into account. Also, sgLASSO relies on two regularization parameters, λ_*S*_ for voxel sparseness and λ_*G*_ for group sparseness, which makes fine parameter tuning and optimization computationally demanding. A preferable procedure would thus be to use gLASSO to achieve good classification performance without heavy computing needs for parameter optimization and use simple thresholding of weights as a practical way to determine the most relevant voxels within selected brain areas. Alternatively, if enough test data is available, sgLASSO can be applied using only voxels of brain areas preselected with gLASSO. This is preferable when handling large datasets.

### gLASSO vs SVM

Both gLASSO and sgLASSO achieved better accuracy and *Fscore* than SVM for semantic task data. Their performance was slightly, but significantly surpassed by SVM for the phonological task and combined data. However, as mentioned above, gLASSO and sgLASSO offer other benefits, which are highly advantageous in clinical practice.

The major advantage is the straight-forward interpretation of weights attributed to features. SVM constructs weights by linear combination of support vector data, which results in non-zero weights for all voxels (Fig 8(d)). This makes it difficult to draw conclusions about brain areas relevant to diagnosis. In contrast, gLASSO as well as sgLASSO reveal discriminating brain areas by reducing the number of voxels to those most relevant. In the case of gLASSO this is done in a group-wise manner, while in the sgLASSO, the number of voxels within groups is reduced as well.

Brain areas contributing to the prediction were in agreement with their known functions and their relationships to verbal fluency and symptoms of depression. The *left precuneus*, for example, is generally interpreted as a hub to the prefrontal lobe [35], a connection that affects motivation, planning, social behavior, and speech production [36]. It is also part of the default mode network, that evidence suggests is altered in depressive patients. Moreover, Krug et al. [37] found in a study with a similar verbal fluency task, that the *left precuneus* was more activated in healthy subjects carrying an allele variant of a specific gene, found to be overrepresented in patients suffering from bipolar disorder, MDD, or schizophrenia. Similarly, the *left precentral gyrus* is explicitly involved in semantic tasks [38], as well as emotion processing [14]. However, the true relation between indicated brain areas and depression remains to be investigated, especially since verbal fluency is impaired in a variety of mental diseases. On the other hand, this implies that the same experiment conducted with patients with other diseases, could reveal either differences or coincidences in the neural origin of common symptoms. The results of such experiments would be of high clinical value and could be revealed by extending the LASSO approach to a multi-class problem.

Another advantage of the LASSO algorithm is its graded, probabilistic prediction. In the presented study, for example, when applying gLASSO or sgLASSO to the combined data, classification was only incorrect for subjects with an absolute normalized discriminative score lower than 0.2 (Fig 6). That means that the diagnosis of a subject as depressed can be considered certain if the normalized discriminative score is higher than 0.2. Similarly, a subject can certainty be considered diagnosed as healthy if the normalized discriminative score is lower than −0.2.

Remarkably, false negative classification occurred only for patients whose condition improved significantly over the following six weeks, so we can assume that patients with normalized discriminative scores < 0.2 are likely to recover. Alternatively, we can define a separate measure of confidence based on the overlap of the two discriminative score distributions. However, these assumptions have to be confirmed using more extensive datasets.

Mwangi et al. [31] as well as Nouretdinov et al. [14] have already drawn attention to the advantage of probabilistic prediction. Mwangi et al. illustrated this by comparing SVM and Relevance Vector Machine, which has an identical functional form to SVM, but uses Baysian inference to achieve probabilistic classification [39]. However, as with SVM, weights are difficult to interpret with respect to the most relevant brain areas. In addition, computation time can increase considerably with increased data size [39].

Nouretdinov et al. propose transductive conformal predictors (TCP, [14, 40]) to provide the classification model with a confidence measure. In contrast to our approach, where the confidence level of a prediction can be deduced from the overlap of discriminative score distributions, TCP provides a confidence level for each prediction based on the relative number of training samples that differ markedly from the test sample in terms of a certain non-conformity measure. However, even though all predictions with a confidence level of 95% were correct, the number of certain predictions (predictions for which the output predictor contained only one label) was 0. This type of dual output is difficult to interpret compared to discriminative score distributions provided by the LASSO method.

Further, TCP is based on pre-selection of voxels via two sample t-test. This step is not necessary in the application of LASSO and prevents potentially useful information from being discarded. The present study demonstrates this effect. Feature selection with a two-sample t-test of semantic verbal fluency data would have left us with no features to which the LASSO algorithm could be applied. Similarly, results of phonological data show that LASSO picked up voxels from brain regions that were not indicated as significantly different in the two-sample t-test (frontal, post central and temporal areas).

### Summary

In summary, we verified that gLASSO and sgLASSO are superior to sLASSO in terms of classification robustness and preferable to the commonly used SVM with respect to feature selection, i.e., identification of relevant brain areas, and probabilistic prediction. The large number of input features is successfully handled without the need of low-dimensional feature extraction. Topographic continuity of non-zero weights can be achieved by adequately grouping input features, thus elucidating discriminative brain areas. Finally, a measure of diagnosis reliability is provided by the discriminative score. We therefore found that gLASSO and sgLASSO are preferable for classification of depression-related fMRI data and identification of relevant brain areas. Further investigation of these brain areas may contribute to the establishment of new prevention and therapeutic programs. This study is the first to explicitly control for data from patients who had not previously been diagnosed with depression.

Sparse classification algorithms for imaging data have independently been considered in two recent studies concerning Alzheimer’s and mild cognitive impairment [41, 42]. Liu et al. [41] raised similar concerns about standard LASSO as presented here and proposed a tree-guided approach combined with SVM to achieve spatial feature grouping. Xin et al. [42] presented a fast, scaleable, generalized, fused LASSO algorithm for the same problem, which could provide an alternative to sgLASSO. However, this remains to be investigated (Sparse group LASSO is also used in studies by Zhou et al. [43], Tsao et al. [44] and [45] but concerns temporal grouping of progressive stages rather than grouping of structural features.).
